# The Survival Rate of the Retention System for Extraoral Maxillofacial Prosthetic Implant: A Systematic Review

**DOI:** 10.7759/cureus.70705

**Published:** 2024-10-02

**Authors:** Uswah Khan, Pankaj Dhawan, Neha Jain

**Affiliations:** 1 Department of Prosthodontics, Manav Rachna Dental College, Faridabad, IND

**Keywords:** craniofacial implants, extraoral implants, facial prosthesis, retention system, survival rate

## Abstract

This systematic review examines the survival rate of retention systems available for extraoral implant-supported prosthesis as there appears to be a lack of information in the literature regarding the same due to the limited data that represents the benefits and drawbacks of each system. The current investigation addresses this gap. If clinicians recognize the optimal survival rate of each retention system, they can select the appropriate retentive attachments increasing patient satisfaction regarding the use craniofacial prosthesis. The objective of this systematic review is to determine the optimal retention system for implant-retained craniofacial prosthesis. This systematic review has followed the Preferred Reporting Items for Systematic Reviews and Meta-Analyses (PRISMA) guidelines. A comprehensive electronic database search was performed to locate research articles on implant-retained craniofacial prostheses published from 2002 to 2024. The included studies were conducted in the English language and specifically compared various retention techniques or provided information on the survival rate, mechanical behavior, and prosthetic complications. Ten of 2857 satisfied the requirements for inclusion and were analyzed.

## Introduction and background

Facial abnormalities can be caused by trauma, congenital illnesses, or ablative tumor surgery affecting the aesthetics, psychological state, social conduct, and quality of life of patients. Such patients can be rehabilitated by either surgical reconstruction or prosthetic rehabilitation [1]. Surgical reconstruction may offer a long-term solution to replace the lost tissue. However, depending on the extent of tissue loss, the availability of donor tissue, the psychophysical state of the patient, and technical difficulties, it is a cumbersome procedure. With none of the risks associated with reconstructive surgery, removable facial prostheses provide both functional and aesthetic benefits [2]. Published research indicates that the best option for treating people with facial deformities is a craniofacial prosthesis. However, it can be difficult to keep craniofacial prostheses in place [3].

Skin adhesives or extraoral implants can be used to achieve retention. Each method has its advantages and disadvantages. Skin adhesives may deteriorate as a result of perspiration or moisture, leading to discomfort for patients during their daily activities. The presence of adhesive residue on the prosthesis can lead to the gradual buildup of skin secretions and the infiltration of bacteria over an extended period of time [4]. Another complexity associated with the utilization of skin adhesives is the occurrence of rough edges due to the repeated application along with the removal of an adhesive-based prosthesis [5]. Collectively, these factors diminish the prosthesis' overall longevity. Craniofacial prostheses are most commonly attached using extraoral implants [6]. This demands a better understanding of the retention system for extraoral implant. There are various retention systems that are used for extraoral implants such as bar and clip, magnets, and O-rings; each system has its own advantages and disadvantages [7]. It becomes critical to determine the retention system that has a better survival rate to reduce undesired side effects such as implant failure, bar or clip fracture, or the loss of magnetic retention. While issues with the retention system's component parts have sporadically been documented, the majority of published research to date has concentrated on implant osseointegration success, as well as soft tissue complication rates. This systematic review aimed to identify the most effective retention system for implant-retained craniofacial prostheses by analyzing the existing data. To the best of our knowledge, there had been no previous comprehensive evaluation of craniofacial prostheses secured by implants using various retention mechanisms.

## Review

Material and methods

The Preferred Reporting Items for Systematic Reviews and Meta-Analyses (PRISMA) guidelines were followed in the conduct of this systematic review [8]. The research question was, which retention system has the highest survival rate for extraoral implant-retained maxillofacial prosthesis? It was registered (ID: CRD42024556426) with the International Prospective Register of Systematic Reviews (PROSPERO) with the following population, intervention, control, outcome, and study design (PICOS) strategy: population (P), patients with facial defects rehabilitated with extraoral implant-retained prosthesis; intervention (I), maxillofacial prostheses retained by various retention systems; control (C), not applicable; outcome (O), survival rates of the various retention systems used for maxillofacial prosthesis; and study design (S), observational studies.

Search strategy

Scopus, PubMed, and Google Scholar databases were searched with the Medical Subject Heading (MeSH) key terms facial prosthesis, extraoral implants, craniofacial implants, and survival rate from 2002 to 2024. Google Scholar was selected because it can identify a large body of grey literature and also cater to all quality articles not just the ones that are published in high-impact journals, thus making Google Scholar an important supplement to other databases, further increasing the comprehensiveness of searches for evidence. Detailed search strategy for each database is described in Table 1. A total of 2857 articles were collected.

**Table 1:** Search strategy

Database	Search strategy	Articles found
PubMed	Extra oral implants OR maxillofacial Rehabilitation OR maxillofacial prosthesis AND retention OR Bar and clip OR magnet retained and survival rate	N=1028
Scopus	survival AND retention AND system AND maxillofacial OR craniofacial OR extraoral AND prosthesis	N=9
Google Scholar	"survival" AND "retention system" AND "maxillofacial" OR "craniofacial" OR "extraoral" AND "prosthesis"	N=1820

Inclusion Criteria

The inclusion criteria were set to include full-text articles that specifically compared various retention techniques or provided information on the survival rate, mechanical behavior, and prosthetic complications of the various retention systems used for extraoral implants. Experimental studies such as randomized controlled trials, comparative studies, observational research such as cohort studies, cross-sectional studies, and case-control studies were also included.

Exclusion Criteria

This review did not consider narrative reviews, individual viewpoints, book chapters, conference papers, case reports, animal experiments, and in vitro studies. Studies evaluating survival rate, mechanical behavior, and prosthetic complications of the various retention systems used for extraoral implants were also excluded. Additionally, studies with incomplete data, those not meeting the inclusion criteria, articles from fields other than dentistry, and articles not in the English language were also excluded.

Mode of Screening

The literature search and screening were carried out by two investigators: UK and NJ. Duplicates were removed manually to ease the process of screening; the titles and abstracts of 1057 articles that remained after duplicate removal were scanned manually. To do this in a streamlined, unbiased way, pre-defined eligibility criteria were followed. The remaining 170 articles were fully read, out of which 160 articles were removed due to various reasons such as they did not evaluate survival rate, mechanical behavior, or prosthetic complications of the various retention systems used for extraoral implants and studies had incomplete data. A senior investigator (PD) was consulted in the event of any disagreement, and a consensus was obtained through debate before a final decision was made.

**Figure 1:**
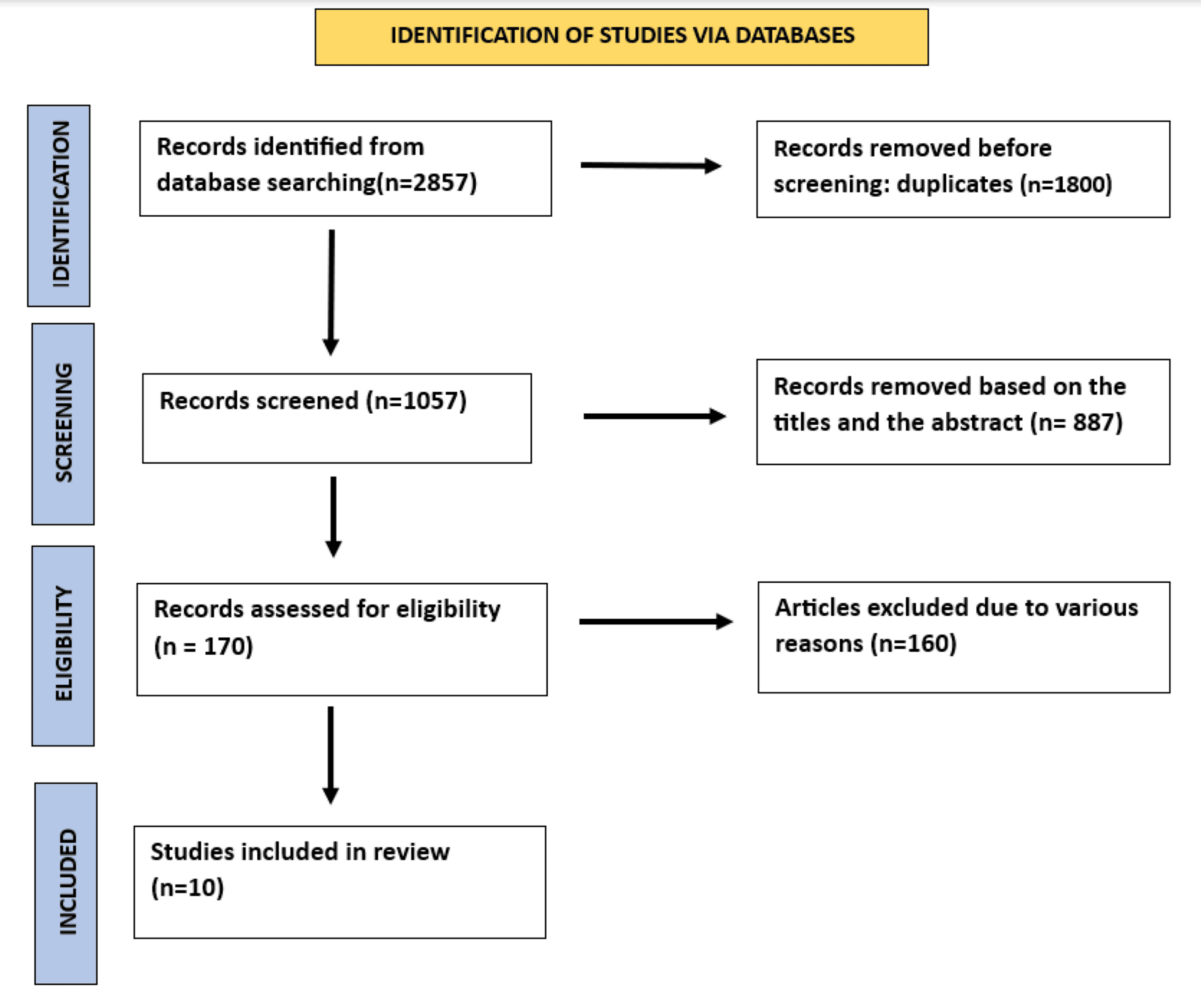
Preferred Reporting Items for Systematic Reviews and Meta-Analyses (PRISMA) flow diagram

A PRISMA flowchart illustrating the process of determining the eligible studies is presented in Figure 1.

Data extraction was meticulously carried out using an Excel spreadsheet (Microsoft Corp., Redmond, WA) and subsequently verified for precision by the senior investigator PD.

Risk of Bias Assessment

Utilizing the National Institutes of Health (NIH) quality assessment tool for a non-randomized clinical appraisal checklist, the quality of the articles was assessed and compiled in Table 2 [9]. It consisted of 14 questions; quality was rated as poor (0-4 out of 14 questions; 0-2 out of nine questions), fair (5-10 out of 14 questions; 3-6 out of nine questions), or good (11-14 out of 14 questions; 6-9 out of nine questions) (NA, not applicable; NR, not reported). The articles were independently scored by two junior investigators: UK and NJ. In case of any disagreement, the senior investigator was consulted, and a final decision was attained by thorough discussions. The ultimate score was determined by calculating the percentage of affirmative responses. Seven selected studies were prospective, and three were retrospective.

**Table 2:** NIH quality assessment tool for non-randomized clinical trials NIH: National Institutes of Health

	Balik et al. (2016) [10]	de Sousa and Mattos (2008) [11]	Curi et al. (2012) [12]	Pekkan et al. (2011) [13]	Sigua-Rodriguez et al. (2017) [14]	Karakoca et al. (2010) [15]	Karakoca et al. (2010) [16]	Karayazgan-Saracoglu et al. (2010) [17]	Roumanas et al. (2002) [18]	Visser et al. (2020) [19]
1. Was the research question or objective in this paper clearly stated?	Yes	Yes	Yes	Yes	Yes	Yes	Yes	Yes	Yes	Yes
2. Was the study population clearly specified and defined?	Yes	Yes	Yes	Yes	Yes	Yes	Yes	Yes	Yes	Yes
3. Was the participation rate of eligible persons at least 50%?	Yes	Yes	Yes	Yes	Yes	Yes	Yes	Yes	Yes	Yes
4. Were all the subjects selected or recruited from the same or similar populations (including the same time period)? Were inclusion and exclusion criteria for being in the study prespecified and applied uniformly to all participants?	Yes	Yes	Yes	Yes	Yes	Yes	Yes	Yes	Yes	Yes
5. Was a sample size justification, power description, or variance and effect estimates provided?	Yes	Yes	Yes	Yes	Yes	Yes	Yes	Yes	Yes	Yes
6. For the analyses in this paper, were the exposure(s) of interest measured prior to the outcome(s) being measured?	No	No	No	No	No	No	No	No	No	No
7. Was the timeframe sufficient so that one could reasonably expect to see an association between exposure and outcome if it existed?	Yes	Yes	Yes	Yes	Yes	Yes	Yes	Yes	Yes	Yes
8. For exposures that can vary in amount or level, did the study examine different levels of the exposure as related to the outcome (e.g., categories of exposure or exposure measured as continuous variable)?	Not mentioned	Not mentioned	Not mentioned	Not mentioned	Not mentioned	Not mentioned	Not mentioned	Not mentioned	Not mentioned	Not mentioned
9. Were the exposure measures (independent variables) clearly defined, valid, reliable, and implemented consistently across all study participants?	Yes	Yes	Yes	Yes	Yes	Yes	Yes	Yes	Yes	Yes
10. Was the exposure(s) assessed more than once over time?	No	No	No	No	No	No	No	No	No	No
11. Were the outcome measures (dependent variables) clearly defined, valid, reliable, and implemented consistently across all study participants?	Yes	Yes	Yes	Yes	Yes	Yes	Yes	Yes	Yes	Yes
12. Were the outcome assessors blinded to the exposure status of the participants?	Not mentioned	Not mentioned	Not mentioned	Not mentioned	Not mentioned	Not mentioned	Not mentioned	Not mentioned	Not mentioned	Not mentioned
13. Was loss to follow-up after baseline 20% or less?	Not mentioned	Not mentioned	Not mentioned	Yes	Not mentioned	Not mentioned	Yes	Not mentioned	Not mentioned	Yes
14. Were key potential confounding variables measured and adjusted statistically for their impact on the relationship between exposure(s) and outcome(s)?	Yes	Yes	Yes	Yes	Yes	Yes	Yes	Yes	Yes	Yes
Quality rating (Good, fair, or poor)	Fair	Fair	Fair	Good	Fair	Fair	Good	Fair	Fair	Good

Results

From the initial search on PubMed, Scopus, and Google Scholar databases, a total of 2857 articles were found, out of which 1057 remained after duplicate removal; further, after title and abstract screening, 170 articles remained. From these articles, 160 articles were removed because they did not fit the inclusion criteria. Ultimately, 10 articles that fulfilled the inclusion criteria were evaluated for quality, which consisted of original research. Seven selected studies were prospective, and three were retrospective. The data collected shows the survival rate of various retention systems. The details of the selected studies are mentioned in Table 3.

**Table 3:** Details of the included studies QoL: quality of life

Serial number	Author	Type of study	Year	Objective	Result
1	Balik et al. [10]	Prospective study	2016	The outcome of the study was that implant survival rate depends on the region its placed due to the density of the bone; also, bar and clip showed better retention	The survival rate of the retention system was bar and clip, followed by magnets
2	de Sousa and Mattos [11]	Prospective study	2008	The retention provided by the bar-clip attachment with three clips remained stronger than that provided by all other systems tested. At the end of the wear test, the magnetic systems showed very little loss of retention but were still less retentive than the bar-clip systems, suggesting higher durability under clinical simulation despite the lower retention initially provided	The retention provided by the bar-clip attachment with three clips remained stronger, hence the improved survival rate. The bar-clip attachment with two clips showed a significant loss of retentive force after wear testing (P < 0.05), suggesting lower durability and shorter clinical life, followed by magnets
3	Curi et al. [12]	Retrospective study	2012	From this study, it was concluded that craniofacial rehabilitation with extraoral implants is a safe, reliable, and predictable method to restore the patient's normal appearance	The two-year overall prosthesis survival rates were 100% for auricular implants, where bar and clip were used as the retention system; 90.0% for nasal implants; and 92.3% for orbital implants, where magnets were used as the retention system
4	Pekkan et al. [13]	Prospective study	2011	Implant patients benefited from bars and magnetic attachments. But in terms of survival rate, bar and clip proved to be superior than magnets	In the bars and clips, the retention system was used in this study, and it showed better survival rate than magnets; also, it was concluded that it has better mechanical advantage. Disadvantages are the cost and the increased difficulty for the patient in performing the necessary implant-associated hygiene
5	Sigua-Rodriguez et al. [14]	Prospective study	2017	The evaluated systems showed that tensile strength for the group retained by the bar-clip system (29.60 N) was higher with statistically significant difference (P < 0.05) when compared to the group retained by the ball/O-ring system (9.41 N) and magnet system (8.61 N)	The tensile strength for the group retained by the bar-clip system (29.60 N) was higher with statistically significant difference (P < 0.05) when compared to the group retained by the ball/O-ring system (9.41 N) and magnet system (8.61 N) for all periods assessed. The ball/O-ring system showed a loss of retention during the fatigue test (Kruskal-Wallis chi-squared = 17.28); hence, it was established that the bar-clip system has better survival rate than other systems
6	Karakoca et al. [15]	Retrospective study	2010	The outcome of this study was that implant-retained extraoral prostheses had limited survival rates. The primary reasons for making new prostheses were discoloration, tearing, and mechanical failures of the acrylic resin substructure or retentive elements. Common complications were the need for clip activation, loosening of bar screws and abutments, and the loss of attachment between silicone and the acrylic resin substructure	The Kaplan-Meier survival estimation method was used. The loss of retention of clips was observed in the patients with bar-and-clip-retained auricular (71%) and nasal (62.5%) prostheses. Clip replacement was required in a number of auricular (9.7%) prostheses due to the dislodgement and fracture of clips. Loosening of prosthetic bar screws was detected in auricular (32.3%) and nasal (50%) groups, with no significant difference. The loss of attachment between the silicone and substructure occurred. In auricular prostheses, this complication occurred with significantly higher frequency (31.1%) than in orbital (8%) and nasal (7.7%) prostheses (P < 0.05)
7	Karakoca et al. [16]	Retrospective study	2010	QoL for patients with maxillofacial defects increased with auricular and nasal prostheses was retained with bar-clip-orbital prostheses than the magnet-retained prosthesis	The degree of satisfaction with auricular and nasal prostheses was significantly greater than with bar-clip-retained orbital prostheses and the magnet-retained prosthesis. It was concluded that the bar and clip retention system has better survival rates that magnets
8	Karayazgan-Saracoglu et al. [17]	Prospective study	2010	The defect area has a significant effect on success rate. The overall success rate was found highest in the auricular area and least in the midfacial area	Prosthesis survival rate increases with bar and clip retention system as compared to magnets
9	Roumanas et al. [18]	Prospective study	2002	Findings indicate Hader clips proved to have better survival rate than other retention systems; also, high survival rates of implants were observed in the auricular and piriform/nasal sites and a less favorable outcome in the orbital region, especially in irradiated sites. Survival rates remained relatively stable after six months for auricular and piriform sites, but a continued downward trend with higher failure rates over time was noted for the orbital region	Hader clips provide a very high level of retention in comparison to magnets, especially in the auricular region, where lateral or sheer forces can easily displace the ear. This is an important consideration for determining the survival rate of retention system
10	Visser et al. [19]	Prospective study	2020	The outcome of this clinical study was that when compared with the bar-clip system, the magnetic retention system showed lower retention forces, hence decreasing the survival rate of the magnetic retention system	When compared with the bar-clip system, no additional aftercare was needed for the magnetic retention system. The lower retention forces of the magnets compared to those of the bar-clip system were a disadvantage, especially for the younger participants in this study

Discussion

This review's objective was to examine the survival rate of various retention systems used in extraoral implants and determine which one had the highest survival rate for a mean period of 2.5-3 years. There are various retention systems that we came across such as the bar-clip retention system, retention with the aid of a magnet, and the ball/O-ring system [14-16].

Over the years, many authors have evaluated retention systems based on patient preference, ease of fabrication, and wear and tear, but there is less data on the survival rate for each system [20]. However, in the year 2008, de Sousa and Mattos evaluated the bar-clip and magnetic systems' survival rates, and due to the magnets' lack of retention, the bar-clip system was considered to have a better retention mechanism and showed better survival rates [11].

Three methods of retention for auricular prostheses were studied by Sigua-Rodriguez et al., the magnetic system, the ball/O-ring system, and the bar-clip system; the retentive strength of each system was found to be 8.61 N, 9.41 N, and 29.60 N, respectively; they concluded that bar and clip had the highest retentive strength and survival rate out of all the systems [14]. However, a combination of mechanical and technical aspects, along with patient-specific factors such as age and manual dexterity, should make up the optimal retention system for the patient.

Recent years have seen the significant availability of new magnets with an extra O-ring lock attachment. These magnets were tested in a study by Visser et al. in 2020, and the results showed improved stability, retention, and survival rates comparable to those of the bar-clip system [19]. Three retentive systems, ball and keeper, bar and clip, and magnet and keeper, were compared in the study done by Pekkan et al. [13]. The study results showed a mechanical advantage through the use of bars and clips, suggesting a better survival rate of the bar and clip system.

In an in vivo study conducted by Williams et al., they compared the Hader bar with clip design to locator devices and found that the locator produced higher peri-implant stresses, thereby decreasing the survival rate of a prosthesis, as well as the implant [21]. Curi et al. also stated that bar-clip retention was employed by 28.6% (16 patients) during physical activity, and it prevented the unintentional displacement of the prosthesis; they concluded that bar and clip have better retentive capability and survival rate, but the authors also stated that magnets generate relatively low-moment forces on the supporting abutments when the prosthesis is in place and withdrawn. Granström et al. also observed a few drawbacks of the bar and clip system; they stated that the utilization of bars and clips connected to implants for prosthesis placement limits usability and implant hygiene while also raising the likelihood of fracture, which significantly impairs the longevity of the implant [22]. In the study conducted by Karakoca et al., the patients who utilized orbital prostheses secured with bars-clips expressed the least satisfaction in regard to cleansing the prostheses [15]. Furthermore, these patients expressed the least amount of satisfaction when it came to the process of placing and removing the prosthesis. However, the scores have been not statistically distinguishable from those of patients who are suffering from nasal and auricular defects.

This study discovered that bar-clip systems have historically been utilized in implant-supported maxillofacial prostheses and have demonstrated superior rates of longevity in comparison to alternative retention systems. Nevertheless, other retention systems such as magnets provide superior hygienic, mechanical, and esthetic benefits. The loss of clip retention was found to be one of the common shortcomings with the bar-retained prosthesis in a retrospective study conducted by Karakoca et al. in the year 2010 wherein they evaluated the survival rates and prosthetic complications associated with implant-retained extraoral prostheses [16]. Loosening of bar screws was also an important finding and is quite a common issue in both auricular (32.3%) and nasal (50%) groups. Another shortcoming noticed in patients wearing bar-retained prostheses was the loss of connection between the silicone and substructure.

Despite having a better survival rate than other retention systems, there were a few shortcomings of the bar and clip retention system. This systematic review may prove to be useful since it can serve as a significant instrument for future research using well-designed methodology to analyze the distinctions between various retention systems used for craniofacial prostheses.

Limitations of the Study

The current systematic review has a few potential limitations due to the fact that only a limited database was searched and limited observational studies were assessed; also, the studies that were assessed mentioned short-term follow-ups of patients and low participant numbers. Despite these limitations, the present systematic review was able to synthesize the evidence gathered from the available studies to analyze the survival rate of the retention system used for extraoral implants and also leaves the scope for additional studies, preferably in the clinical trial form required to validate these outcomes. Meta-analysis was not performed due to statistical heterogeneity.

## Conclusions

It can be concluded that bar-clip provides better retention and has better survival rates than magnetic-retained auricular prosthesis; both retention systems have their drawbacks and advantages. Nevertheless, it was discovered that the magnets were more sanitary compared to the bar and clip. Hence, it can be inferred that the choice between a bar-clip or magnetic-retained auricular prosthesis is a personal decision. Both systems are effective treatment options with their own advantages along with their limitations.
